# CTLA-4 rs231775 and risk of acute renal graft rejection: an updated meta-analysis with trial sequential analysis

**DOI:** 10.1038/s41598-020-69849-4

**Published:** 2020-07-30

**Authors:** Sarah Cargnin, Ubaldina Galli, Jae Il Shin, Salvatore Terrazzino

**Affiliations:** 1 Department of Pharmaceutical Sciences and Interdepartmental Research Center of Pharmacogenetics and Pharmacogenomics (CRIFF), University of Piemonte Orientale, Largo Donegani 2, 28100 Novara, Italy; 2 Department of Pharmaceutical Sciences, University of Piemonte Orientale, Novara, Italy; 3 Department of Pediatrics, Yonsei University College of Medicine, Seoul, Republic of Korea

**Keywords:** Genetics, Immunology, Biomarkers, Molecular medicine

## Abstract

Contrasting results exist on the association between CTLA-4 rs231775 and acute rejection in kidney transplant recipients. We herein conducted an updated systematic review with meta-analysis and trial sequential analysis (TSA) to clarify this relationship and to establish whether the current evidence is sufficient to draw firm conclusions. In addition, noteworthiness of significant pooled odds ratios (ORs) was estimated by false positive report probability (FPRP). A comprehensive search was performed through PubMed, Web of Knowledge, Cochrane Library and Open Grey up to October 2019. Fifteen independent cohorts, including a total of 5,401 kidney transplant recipients, were identified through the systematic review. Overall, no association was detected with the allelic (OR 1.07, 95% CI 0.88–1.30, P = 0.49), dominant (OR 0.94, 95% CI 0.73–1.22, P = 0.66) or the recessive (OR 1.18, 95% CI 0.97–1.43, P = 0.096) model of CTLA-4 rs231775. In each genetic model, the cumulative Z-curve in TSA crossed the futility boundary and entered the futility area. In addition, none of the significant genetic comparisons detected in the subsequent and sensitivity analyses or in previously reported meta-analyses were found to be noteworthy by FPRP. In conclusion, this study provides strong evidence that CTLA-4 rs231775 is not a clinically-relevant genetic risk determinant of acute rejection after renal transplantation.

## Introduction

Cytotoxic T lymphocyte antigen-4 (CTLA-4, also known as CD152) is a transmembrane homodimer glycoprotein expressed by activated effector T cells (Teffs) that negatively regulates T cell-mediated immune responses^1^. CTLA-4 exerts its immunosuppressive function through a variety of mechanisms which include competition with the co-stimulatory CD28 molecule for binding to their shared B7 ligands (CD80/CD86) on the antigen-presenting cells (APC)^2^, and interference with TCR-mediated signal transduction^3^. Given the key role of CTLA4 on regulation of allograft rejection and tolerance^4,5^, a great attention has been focused on the relationship between CTLA-4 genetic variation and graft outcome following solid organ transplantation^6–9^.

The human CTLA-4 gene is located on the short arm of the second chromosome (2q33) and consists of four exons encoding respectively the leader sequence peptide, an extracellular immunoglobulin like domain containing the binding site, a transmembrane domain and a cytoplasmic tail^10,11^. The single-nucleotide polymorphism (SNP) rs231775 of the *CTLA*-*4* gene, also referred as + 49A>G, causes a threonine-to-alanine substitution at codon 17 in the peptide leader sequence, and guanine at this position is related to reduced CTLA-4 protein expression^12^. Over the last fifteen years, several efforts have been made to evaluate the impact of CTLA-4 rs231775 on the risk of acute rejection in kidney transplant recipients, however the results differed among studies, which reported a lack of association^13,14^ or a higher risk of acute renal rejection among GG carriers^15,16^ or inconclusive findings because of low sample size^17–19^. The reasons for these conflicting and inconclusive results may be the different ethnicity, the clinical heterogeneity, the low statistical power or a combination of these factors.

Up to now, six meta-analyses have been conducted to clarify the role of CTLA-4 rs231775 on the risk of acute renal graft rejection^20–25^. However, all these studies did not take into account the risk of random errors due to sparse data and multiple meta-analytic up-dates^26,27^, which often result in false positive (type-1 error) and false negative (type-2 error) findings. Given that the above-mentioned issues can be addressed by application of trial sequential analysis (TSA) to meta-analytic results^28^ and the recent publication of two novel primary studies^29,30^, we herein conducted an updated meta-analysis with TSA to assess reliability of the accumulated evidence on the relationship between CTLA-4 rs231775 and acute renal transplant rejection. In addition, noteworthiness of significant pooled estimates from the present and previous meta-analyses was estimated by false positive report probability (FPRP).

## Materials and methods

### Literature search and selection criteria

This systematic review was conducted in accordance with the PRISMA Statement principles^31^. A computerized literature search was carried out on PubMed, Web of Knowledge, Cochrane Library and Open Grey (last search up October 17th, 2019) by using the Boolean combinations of the key terms: (cytotoxic T-lymphocyte antigen 4 OR CTLA4 OR CTLA-4 OR GWAS OR genome-wide association study) AND (polymorphism OR polymorphisms OR SNP OR SNPs OR genotype OR genotypes OR allele OR alleles OR variant OR variants) AND (kidney OR renal) AND rejection. Eligible studies were required to meet the following inclusion criteria: (i) investigating the association between CTLA-4 rs231775 and acute rejection (AR) in kidney transplant recipients (KTRs) (ii) reporting sufficient data for estimating an odds ratio (OR) with 95% confidence interval (CI) for the association with CTLA-4 rs231775. Exclusion criteria were: not human studies or not related to the research topics; case reports, editorials and meeting abstracts; narrative reviews, systematic reviews with or without meta-analysis; duplication of previous publications. The potentially relevant articles were then read in their entirety to assess their appropriateness for inclusion in the systematic review. Reference lists of retrieved studies were also checked to identify other potentially eligible studies. If two or more studies shared part of the same patients’ population, the one with the larger sample size or more complete data was included. The corresponding authors were contacted by e-mail when the eligible paper had insufficient information for calculation of OR and 95% CI. Studies were excluded if the corresponding author did not answer to the e-mail or was unable to provide the requested data.

### Data extraction and study quality assessment

From each identified study the following data were extracted: name of first author, year of publication, study location, ethnicity, mean age, male/female ratio, donor type (i.e. living or deceased), immunosuppressive drugs, criteria for diagnosis of acute rejection, number of KTRs with and without AR, timing of AR after kidney transplantation, method of CTLA-4 rs231775 genotyping, and allele/genotype counts. Methodological study quality was assessed using the Newcastle–Ottawa scale (NOS) for cohort studies (available at: https://www.ohri.ca/programs/clinical-epidemiology/oxford-asp), which consists of three components: (I) selection and definition of the study groups (0–4 points); (II) comparability of the cohorts (0–2 points); and (III) ascertainment of outcomes (0–3 points). Studies with a NOS score ≥ 7 out of 9 were considered of higher quality. All studies were independently analyzed by two reviewers (S.T. and S.C.) and any discrepancies in study selection, data extraction and methodological quality evaluation were resolved through consensus.

### Data synthesis and analysis

For each study, the Hardy–Weinberg Equilibrium (HWE) was calculated using the Pearson’s goodness-of-fit chi-square test implemented in the online Finetti’s program (available at https://ihg.gsf.de/cgi-bin/hw/hwa1.pl). ORs were pooled based on the allelic (G vs. A), dominant (GG/AG vs. AA) or recessive (GG vs. AG/AA) genetic contrast of CTLA-4 rs231775 by using the random-effects (DerSimonian–Laird method) model, which takes into account both within study variance and cross-study variance^32^. In case of lack of heterogeneity, the random effects model coincides with the fixed-effect model^33^. Between-study heterogeneity was tested using the Q statistic, with a p-value < 0.10 indicating the presence of significant heterogeneity among studies. Heterogeneity was also quantified by the I^2^ metric, with I^2^ values > 50% indicating high heterogeneity^34^. The robustness of overall estimates was verified by conducting subgroup and sensitivity analyses. The presence of publication bias or a difference between small and large studies (’small-study effects’) in the overall analyses was evaluated graphically by drawing funnel plots and statistically by means of Egger’s test^35^. In case of statistical evidence of funnel plot asymmetry (P-value of the Egger’s test < 0.10), the ‘trim-and-fill’ method was used to adjust the overall pooled estimate for potential publication size or small study effects^36^. All analyses were performed using ProMeta software (version 2; Internovi di Scarpellini, Daniele SAS, Cesena, Italy) and the significance of pooled ORs was set at P < 0.05. Noteworthiness of significant pooled ORs was also estimated by false positive report probability (FPRP)^37^, which is calculated based on the statistical power of the test, the observed *P-*value, and a given prior probability for the association. FPRP values were calculated at the prior probability of 0.001 (expected for a candidate gene)^38^ to detect ORs of 1.50 (or its reciprocal 1/1.5 = 0.67), by using the Excel spreadsheet provided by Wacholder et al.^39^. A significant result (P < 0.05) with an FPRP value of less than 0.2 indicated a noteworthy association.

### Trial sequential analysis

Trial sequential analysis (TSA) allows to control the risk of type I (false positive) and type II (false negative) errors of conventional meta-analysis and to calculate the required information size (RIS), that is the required number of participants in a reliable and conclusive meta-analysis^27^. We estimated the RIS based on an overall 5% risk of a type I error (two sided α = 0.05), a statistical test power of 80% (β = 0.2) and an “a priori” relative risk difference (reduction or increase) of 15%. In addition, we set the event proportion in the reference genotype or allele group as the median value across studies included in the meta-analysis, and we adjusted the required information size for study heterogeneity by applying a D^2^ adjustment factor^39^. If the cumulative Z curve crosses the trial sequential monitoring boundaries with achievement of RIS, it means that a sufficient level of evidence has been reached and further studies are unneeded. When the Z curve does not cross any of the boundaries and the RIS has not been reached, it can be concluded that more studies are required to reach a sufficient conclusion. If the cumulative Z curve crosses the futility boundaries, the conclusion of indiscrimination between two groups is accepted under the given conditions^27,40^. TSA was performed using Trial Sequential Analysis software^41^ version 0.9.5.10 beta (available at www.ctu.dk/tsa).

## Results

### Literature screening process and characteristics of the identified studies

**Figure 1:**
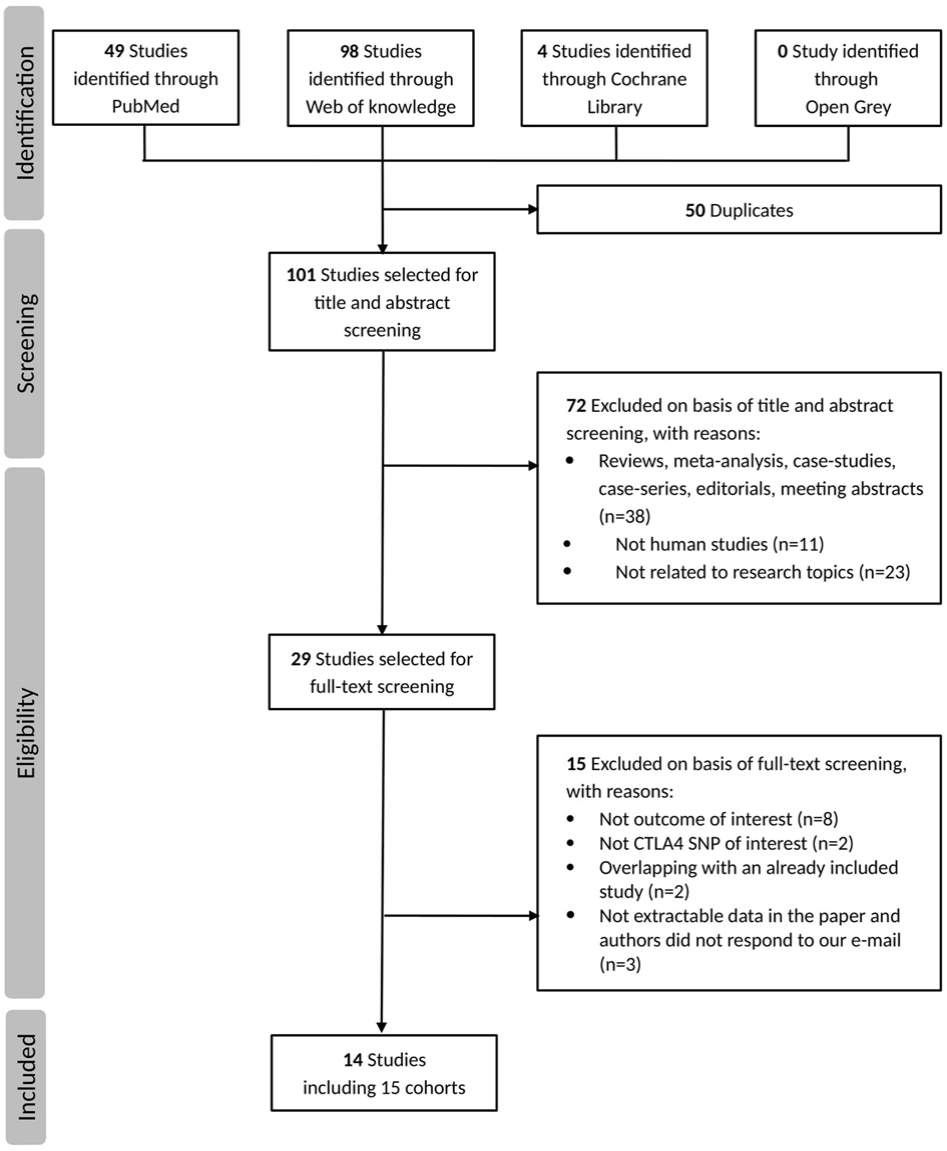
Flow chart of literature search and selection process of eligible studies.

The flow chart illustrating the overall literature selection process is illustrated in Fig. 1. Briefly, the literature search on PubMed, Web of Knowledge, Cochrane Library and Open Grey resulted in a total of 151 citations. After removing of 50 duplicated records, the remaining 101 studies were evaluated by carefully reading of titles, abstracts and full texts. After exclusion of additional 87 not relevant papers, 14 studies describing a total of 15 cohorts were included in the systematic review of association between CTLA-4 rs231775 and risk of acute renal graft rejection^13–19,29,30,42–46^. The main characteristics of the identified studies are summarized in Table 1. In brief, studies were published between 2005 and 2019, mean age ranged from 30 to 49.6 years, and sample size varied from 63 to 2,872. The most represented ethnic populations were Caucasian, Asian and African, which were included, respectively, in eight^13,14,18,19,30,42,44,46^, three^15,16,45^ and two studies^30,43^. The largest study^30^ included a cohort of European Americans (n = 2,390) and a cohort of African Americans (n = 482). In the majority of the identified studies, patients received renal allografts from living or cadaveric donors, while in 4 studies the transplanted kidney came exclusively from a cadaveric donor^13–15,18^.

**Table 1:** Characteristics of studies included in the meta-analysis of association between CTLA-4 rs231775 and acute renal allograft rejection.

First author ^ref^	Year	Location (ethnicity)	Case number (AR/NAR)	Age (year, mean ± SD/range)	Male/female	Donor type (living/deceased)	Timing of AR after KT	P_value_ for HWE	NOS score
Dmitrienko^42^	2005	Canada (Caucasian)	100 (50/50)	44 ± 10.5	57/43	40/59	≤ 1 year	0.04	8
Gendzekhadze^17^	2006	Venezuela (Latino)	63 (30/33)	40 ± 10	37/26	33/30	≤ 3 months	0.51	6
Gorgi^43^	2006	Tunisia (African)	70 (31/39)	30 (12–56)	44/26	–	–	0.18	3
Wiśniewski^44^	2006	Poland (Caucasian)	91 (38/53)	–	–	–	≥ 6 months	0.06	6
Haimila^13^	2009	Finland (Caucasian)	678 (109/535)	49.6 (17.8–74.5)	430/248	0/678	–	NC	8
Kim^45^	2010	Korea (Asian)	325 (59/266)	40.1 ± 11.4	198/127	–	≤ 6 months	0.56	8
Kusztal^46^	2010	Poland (Caucasian)	314 (102/212)	41.9 ± 12.1	184/130	2/312	≥ 5 years	NC	7
Domański^14^	2012	Poland (Caucasian)	269 (70/199)	47.6 ± 13.0	166/103	0/269	≤ 5 years	0.30	7
Gao^15^	2012	China (Asian)	167 (45/122)	46.8 ± 11.3	105/62	0/167	≤ 6 months	0.77	8
Canossi^18^	2013	Italy (Caucasian)	72 (37/35)	–	–	0/72	≥ 6 months	0.67	4
Misra^16^	2014	India (Asian)	190 (36/154)	–	–	–	≤ 3 months	0.006	7
Ruhi^19^	2015	Turkey (Caucasian)	81 (34/47)	35.8 ± 11.7	62/19	81/0	≤ 6 months	0.74	8
Niknam^29^	2017	Iran (Iranian)	172 (45/127)	38.3 ± 14.3	113/59	73/99	≥ 3 months	0.02	7
Oetting^30^	2019	USA (European Americans)	2,390 (421/1969)	50.4 ± 14.7	1,500/890	1617/773	> 12 months	0.84^EA^	9
		(African Americans)	482 (71/411)	46.9 ± 12.2	303/179	155/327	≤ 12 months	0.35^AA^	

*AA* subgroup of African Americans; *AR* acute rejection group; *EA* subgroup of European Americans; *HWE* Hardy–Weinberg Equilibrium; *KT* kidney transplantion; *NAR* no acute rejection group; *NOS* Newcastle–Ottawa scale.

CTLA-4 rs231775 was found in Hardy–Weinberg equilibrium (HWE) in 9 studies^14–19,30,43–45^; it significantly deviated from HWE in three studies^16,29,42^, while it was not computable in two studies^13,46^ which reported genotype data as a combined group (AG/AA vs. GG). The distribution of CTLA-4 rs231775 genotypes for each included cohort and timing of AR assessment after kidney transplantation is shown in Table 2. In seven studies^15–17,19,30,42,45^ the association of CTLA-4 rs231775 with AR was assessed during the first year post-transplant. Other study characteristics, including immunosuppressive drugs, criteria used for acute rejection diagnosis, and the genotyping method of CTLA-4 rs231775 are reported in Supplementary Material, Table S1. With regard to the study quality, the overall NOS scores ranged from 3 to 9 (median 7) (Table 1). Ten studies with a NOS score ≥ 7 were considered of higher quality^13–16,19,29,30,42,45,46^. Individual scores for each item of NOS in the identified studies are shown in Supplementary Material, Table S2.

**Table 2:** Distribution of CTLA-4 rs231775 among the 15 identified cohorts.

First author^ref^ Year	Ethnicity	Time considered after KT	AR				NAR			
			AA	AG	GG	Total	AA	AG	GG	Total
Dmitrienko^42^ 2005	Caucasian	≤ 1 Y	18	29	3	50	23	24	3	50
Gendzekhadze^17^ 2006	Latino	≤ 3 M	9	16	5	30	17	11	5	33
Gorgi^43^ 2006	African	–	7	10	14	31	2	15	22	39
Wiśniewski^44^ 2006	Caucasian	≥ 6 M	13	13	12	38	19	23	11	53
Haimila^13^ 2009	Caucasian	–	37	–	–	109	151	–	–	535
Kim^45^ 2010	Asian	≤ 6 M	6	19	34	59	27	115	124	266
Kusztal^46^ 2010	Caucasian	≥ 5Y	22	–	–	102	48	–	–	212
Domański^14^ 2012	Caucasian	≤ 5Y	22	35	13	70	51	96	32	179
Gao^15^ 2012	Asian	≤ 6 M	4	16	25	45	16	62	44	122
Canossi^18^ 2013	Caucasian	≥ 6 M	18	11	5	34	11	21	2	34
Misra^16^ 2014	Asian	≤ 3 M	14	12	10	36	78	56	20	154
Ruhi^19^ 2015	Caucasian	≤ 6 M	13	17	4	34	23	20	4	47
Niknam^29^ 2017	Iranian	≥ 3 M	28	12	5	45	71	43	13	127
Oetting^30^ 2019	Caucasian	> 12 M	186	176	59	421	704	957	308	1969
Oetting^30^ 2019	African	> 12 M	30	30	11	71	147	189	70	406
Oetting^30^ 2019	Caucasian	≤ 12 M	157	139	49	345	733	994	318	2045
Oetting^30^ 2019	African	≤ 12 M	23	19	11	53	154	200	70	424

*AR* acute rejection group; *KT* kidney transplantation; *M* months; *NAR* no acute rejection group; *Y* year.

### Quantitative data synthesis

**Figure 2:**
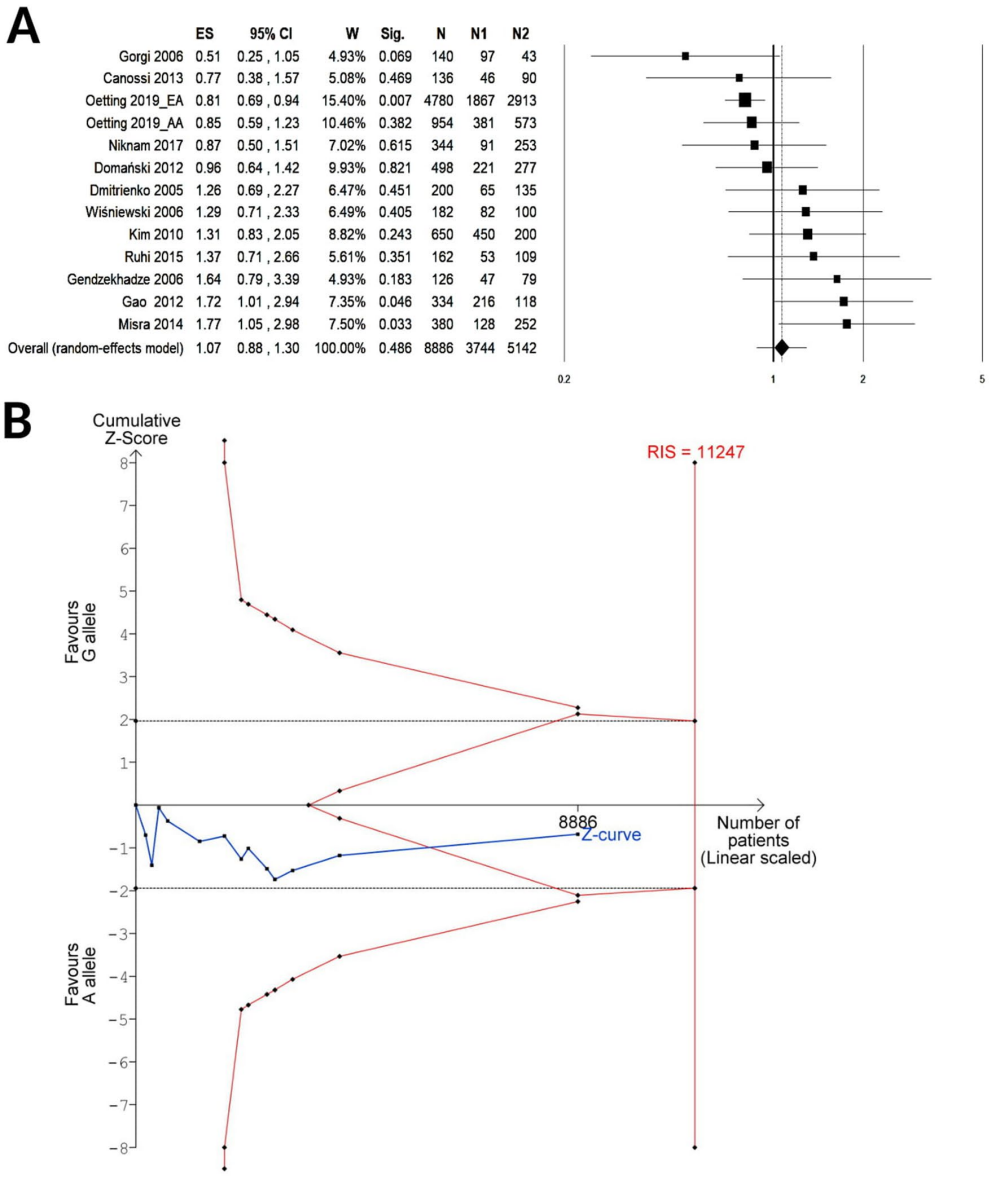
Forest plot (**A**) and TSA (**B**) for the association between the allelic (G vs A) genetic model of CTLA-4 rs231775 and acute renal graft rejection. ES, effect size (i.e. odd ratio); *W* weight; *Sig* statistical significance; *N* total number of alleles; *N1* number of G alleles; *N2* number of A alleles. The required information size (RIS) was calculated based on a two side α = 5%, β = 20% (power 80%), and an “a priori” relative risk increase of 15%.

**Figure 3:**
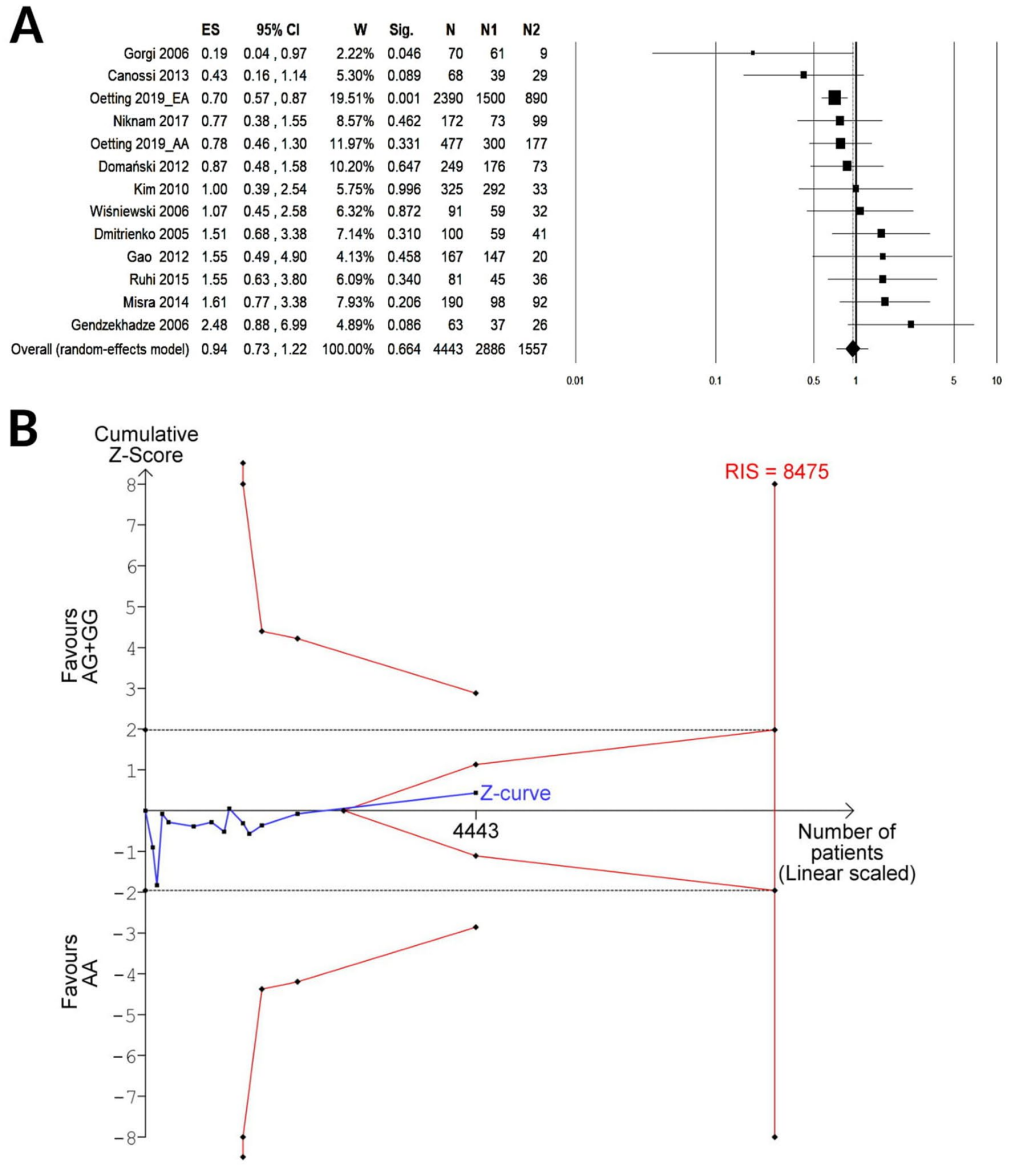
Forest plot (**A**) and TSA (**B**) for the association between the dominant (GG + AG vs AA) genetic model of CTLA-4 rs231775 and acute renal graft rejection. *ES* effect size (i.e. odd ratio); *W* weight; *Sig* statistical significance; *N* total number of kidney transplant recipients; *N1* number of patients with GG or AG genotype; *N2* number of patients with AA genotype. The required information size (RIS) was calculated based on a two side α = 5%, β = 20% (power 80%), and an “a priori” relative risk reduction of 15%.

**Figure 4:**
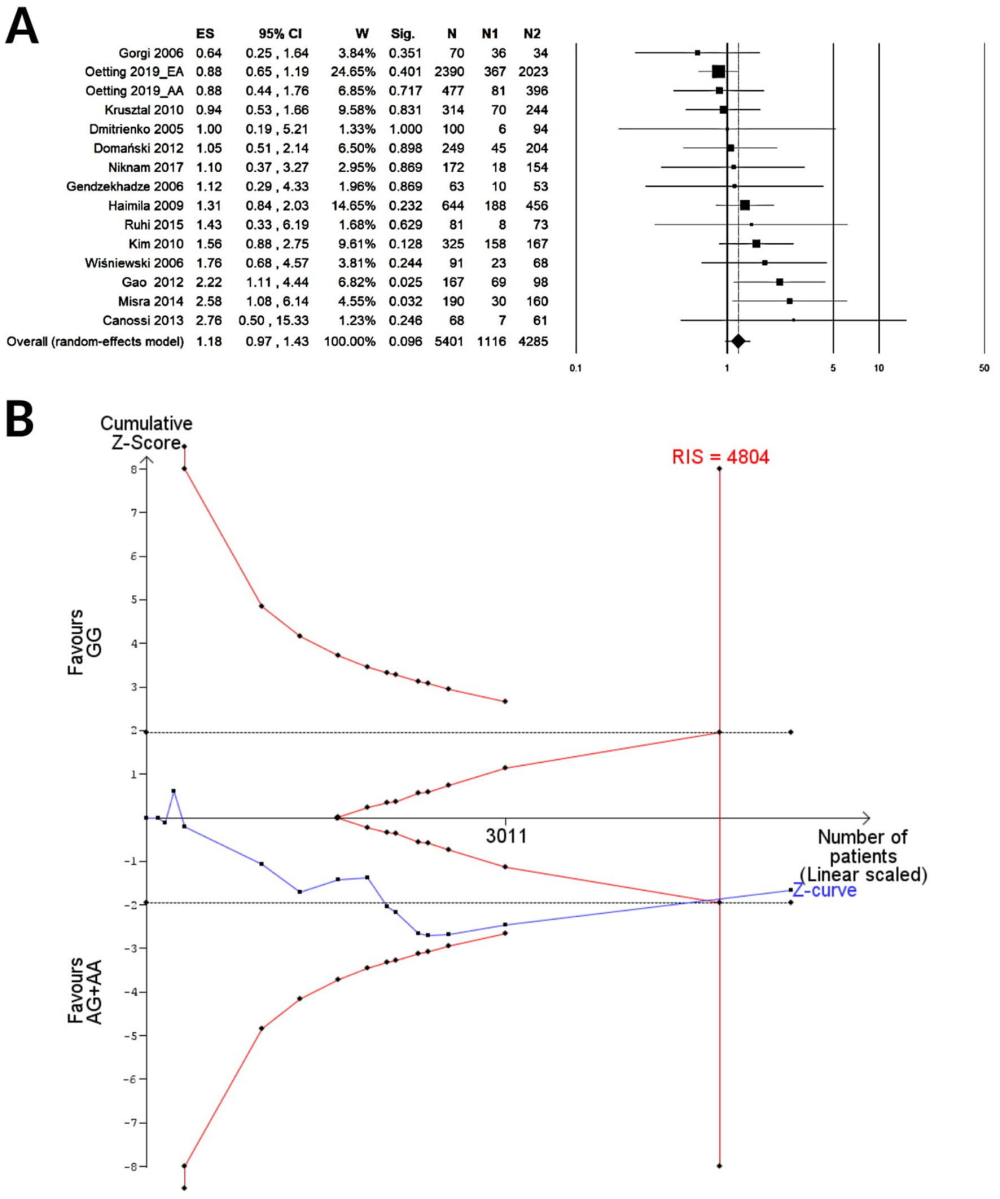
Forest plot (**A**) and TSA (**B**) for the association between the recessive (GG vs AG + AA) genetic model of CTLA-4 rs231775 and acute renal graft rejection. ES, effect size (i.e. odd ratio); W, weight; Sig, statistical significance; N, total number of kidney transplant recipients; N1, number of patients with GG genotype; N2, number of patients with AG or AA genotype. The required information size (RIS) was calculated based on a two side α = 5%, β = 20% (power 80%), and an “a priori” relative risk increase of 15%.

A total of 15 cohorts including 5,401 KTRs were available for the meta-analysis of CTLA-4 rs231775 under the recessive contrast model (GG vs. AG/AA), while 13 cohorts enrolling 4,443 KTRs were available for the allelic (G vs. A) or the dominant (GG/AG vs. AA) model. A summary of random meta-analyses on the effect of CTLA-4 rs231775 on the risk of acute renal graft rejection is shown in Table 3. The pooled results showed no association with the allelic (G vs. A, OR 1.07, 95% CI 0.88–1.30, P = 0.49; Fig. 2A), dominant (GG/AG vs. AA, OR 0.94, 95% CI 0.73–1.22, P = 0.66; Fig. 3A) or the recessive genetic model (GG vs. AG/AA, OR 1.18, 95% CI 0.97–1.43, P = 0.096; Fig. 4A). The TSA for the allelic (Fig. 2B) or the dominant (Fig. 3B) model showed that the cumulative Z-curve (blue line) crossed neither the trial sequential monitoring boundaries (red inward slash), nor the conventional boundaries (black dotted line); however, it entered the futility area without reaching the required information size (G vs. A, RIS = 11,247; GG/AG vs. AA, RIS = 8,475). For the recessive model contrast (GG vs. AG/AA, Fig. 4B), the cumulative Z-curve crossed the futility boundary and reached the required information size (RIS = 4,804).

**Table 3:** Summary of random-effect meta-analyses for the relationship between CTLA-4 rs231775 and acute renal graft rejection.

Group or subgroup	No of cohorts	AR/NAR (genotypes or alleles)	Test of association		Test of heterogeneity		Egger’s P-value	FPRP value at prior probability of 0.001	
			OR (95% CI)	P-value	I^2^ (%)	P-value		Power*	OR = 1.5
**G vs. A**									
Overall	13	1928/6,958	1.07 (0.88–1.30)	0.49	52	0.014	0.045		
Caucasian	6	1,294/4,664	0.93 (0.77–1.12)	0.47	19	0.47			
Asian	3	280/1,084	1.55 (1.16–2.07)	0.003	0	0.62		0.412	0.879
African	2	204/890	0.73 (0.46–1.15)	0.17	33	0.22			
HWE	10	1666/6,296	1.02 (0.83–1.26)	0.89	51	0.030			
NOS ≥ 7	9	1662/6,640	1.10 (0.89–1.36)	0.39	58	0.016			
AR during the first year after KT	7	1,214/6,038	1.17 (0.89–1.55)	0.26	63	0.013			
**GG/AG vs. AA**									
Overall	13	964/3,479	0.94 (0.73–1.22)	0.66	41	0.060	0.14		
Caucasian	6	647/2,332	0.87 (0.63–1.20)	0.40	36	0.16			
Asian	3	140/542	1.38 (0.82–2.32)	0.22	0	0.71			
African	2	102/445	0.47 (0.13–1.80)	0.27	62	0.10			
HWE	10	833/3,148	0.87 (0.65–1.16)	0.35	38	0.10			
NOS ≥ 7	9	831/3,320	0.92 (0.73–1.17)	0.50	26	0.21			
AR during the first year after KT	8	652/3,141	1.13 (0.78–1.65)	0.51	57	0.024			
**GG vs. AG/AA**									
Overall	15	1,175/4,226	1.18 (0.97–1.43)	0.096	11	0.33	0.16		
Caucasian	8	858/3,079	1.04 (0.85–1.28)	0.69	0	0.66			
Asian	3	140/542	1.93 (1.31–2.86)	0.001	0	0.57		0.105	0.909
African	2	102/445	0.79 (0.45–1.38)	0.40	0	0.59			
HWE	10	833/3,148	1.16 (0.89–1.51)	0.26	21	0.25			
NOS ≥ 7	11	1,042/4,067	1.19 (0.96–1.48)	0.11	20	0.25			
AR during the first year after KT	8	652/3,141	1.37 (1.00–1.88)	0.048	30	0.19		0.706	0.985

*AR* acute rejection group; *HWE* Hardy–Weinberg equilibrium; *KT* kidney transplantation; *NAR* no acute rejection group; *NOS* Newcastle–Ottawa scale.

*Power to detect a noteworthy finding by false positive report probability (FPRP) when the true OR equals the specified value.

### Publication bias

**Figure 5:**
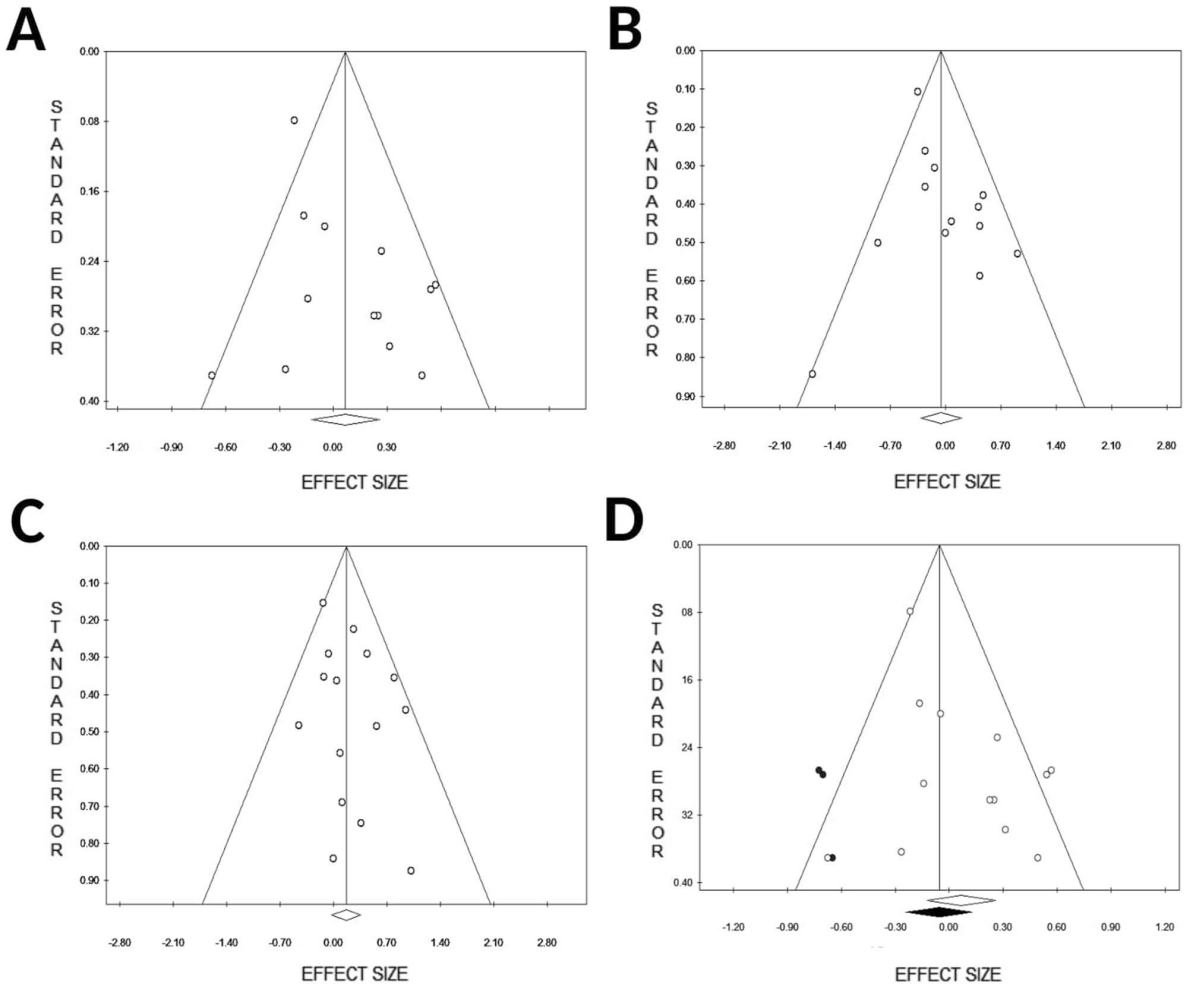
Funnel plots for the association between CTLA-4 rs231775 and acute renal transplant rejection. (**A**) Allelic contrast: G vs A (Egger’s P-value = 0.045). (**B**) Dominant contrast: GG/AG vs. AA (Egger’s P-value = 0.14). (**C**) Recessive contrast: GG vs. AG/AA (Egger’s P-value = 0.16). (**D**) Trim-and-fill funnel plot for the allelic contrast model (G vs. A, adjusted OR 0.95; 95% CI 0.78–1.15, P = 0.57).

In overall analyses, no evidence of publication bias or small study effects was found for the GG/AG vs. AA model (Egger’s P-value = 0.14, Fig. 5B) and the GG vs. AG/AA model (Egger’s P-value = 0.16, Fig. 5C). Conversely, a statistically significant funnel plot asymmetry was detected for the G vs. A model (Egger’s P-value = 0.045, Fig. 5A). The trim-and-fill method for the allelic contrast model (G vs. A) imputed three missing studies on the left side of the funnel plot (Fig. 5D), however the adjusted effect size was still not significant (OR 0.95; 95% CI 0.78–1.15, P = 0.57).

### Subgroup and sensitivity analyses

The Q-statistic indicated the presence of between-study heterogeneity in the meta-analysis for the allelic (P = 0.014, I^2^ = 52%) and dominant (P = 0.060, I^2^ = 41%) models (Table 3), but not for the recessive model contrast (P = 0.33, I^2^ = 11%). In order to explore possible reasons for the observed heterogeneity, we conducted a subgroup analysis stratified by ethnicity, as well as three sensitivity analyses by using the following inclusion criteria: higher study quality (NOS ≥ 7), conformation with HWE, and AR within one year after kidney transplantation. A significant association was detected among Asians for the allelic (G vs. A, OR 1.55, 95% CI 1.16–2.07, P = 0.003) or the recessive model (GG vs. GA/AA, 1.93, 95% CI 1.31–2.86, P = 0.001) in absence of between-study heterogeneity (I^2^ = 0%), while no association with CTLA-4 rs231775 was found among Caucasian or African patients. Results of sensitivity analyses provided evidence of a higher risk of acute rejection in the first year after kidney transplantation among carriers of the rs231775GG genotype (OR 1.37, 95% CI 1.00–1.88, P = 0.048), while no associations were detected among studies conforming to HWE or with a higher quality score (NOS ≥ 7) (Table 3).

### FPRP analysis

Statistically significant findings (P < 0.05) of subgroup and sensitivity analyses were further investigated by using the FPRP test. At the pre-specified prior probability level of 0.001 to detect ORs of 1.50 (or its reciprocal 1/1.5 = 0.67), the FPRP values for the association of rs231775G or rs231775GG among Asians were 0.879 and 0.909, respectively, while the FPRP value for the association of rs231775GG with AR in the first year after kidney transplantation was 0.985 (Table 3). Therefore, none of these associations was found noteworthy under FPRP (cutoff value < 0.2), indicating no reliable results. Similarly, analysis of significant genetic comparisons of previous meta-analyses on the association of CTLA-4 rs231775 and acute renal transplant rejection revealed FPRP values higher than 0.2, indicating lack of noteworthy results (Table 4).

**Table 4:** Significant genetic comparisons from previous meta-analyses evaluating the association between CTLA-4 rs231775 and acute renal graft rejection: results of the FPRP test.

First Author ^ref^, year	Comparison	Overall or subgroup	Sample size	Test of association		Test of heterogeneity		Egger’s P-value	FPRP value at prior probability of 0.001	
				Model/OR (95%CI)	P-value	I^2^ (%)	P-value		Power*	OR = 1.5
Duan^21^, 2012	A vs. G	All	2,208	F/0.805 (0.677–0.957)	0.014	0	0.961	0.508	0.984	0.934
Han^22^, 2014	GG vs. AG/AA	All	2032	F/1.30 (1.00, 1.69)	0.05	5	0.4	0.721	0.857	0.983
Gao^23^, 2015	G vs. A	All	1805	F/1.21(1.03, 1.42)	0.02	19	0.28	0.204	0.996	0.952
		Asian	492	F/1.47 (1.04, 2.07)	0.03	0	0.44	NC^b^	0.546	0.980
	GG/AG vs. AA	Asian	492	F/1.79 (1.15, 2.78)	0.009	0	0.44	NC^b^	0.216	0.978
		African	70	F/0.19 (0.04, 0.97)	0.046	NC^a^	NC^a^	NC^b^	0.066	0.999
	GG vs. AG/AA	All	1,805	F/1.35 (1.05, 1.73)	0.02	0	0.69	0.021	0.797	0.957
Liu^24^, 2017	AA/AG vs. GG	All	2,443	F/0.79 (0.63, 0.98)	0.034	0	0.510	0.39	0.939	0.972
Yang^25^, 2017	G vs. A	All	1,623	F/1.21 (1.03, 1.44)	0.02	26	0.19	NR	0.992	0.970
		Asian	682	F/1.55 (1.16, 2.06)	0.003	NR	0.62	NR	0.411	0.860
	GG vs. AG/AA	All	2,581	F/1.37 (1.10, 1.69)	0.004	NR	0.52	NR	0.801	0.804
		Asian	682	F/1.91 (1.29, 2.84)	0.001	NR	0.57	NR	0.116	0.923

*F* Fixed-effect model, *NR* not reported.

^a^NC, not calculable because of less than two studies.

^b^NC, not calculable because of less than three studies.

*Power to detect a noteworthy finding by false positive report probability (FPRP) when the true OR equals the specified value.

## Discussion

In the two most recent meta-analyses^24,25^, a higher risk of acute rejection has been reported in kidney transplant recipients under the GG vs. AA/AG model of CTLA-4 rs231775, but only the larger study^25^ also detected a higher risk under the G vs. A model contrast. Given the recent publication of two novel primary studies on the risk of acute kidney transplant rejection^29,30^, we conducted an updated meta-analysis with trial sequential analysis (TSA) to better estimate the impact of CTLA-4 rs231775 and to determine whether the currently available evidence was sufficient and conclusive. In addition, false-positive report probability (FPRP) analysis was conducted to examine whether the significant findings of the present or previous meta-analyses were noteworthy. To our knowledge, Dong and colleagues, in 2008, first applied FPRP to assess noteworthiness of meta-analytic estimates in a field synopsis^47^. Since then, more than 40 meta-analyses of genetic association studies have been published in which the FPRP method was applied to examine noteworthiness of significant pooled estimates.

This updated systematic review and meta-analysis, which included a total of 5,401 kidney transplant recipients, showed that CTLA-4 rs231775 is not a genetic determinant of acute rejection. Results from the traditional pooled analysis technique are corroborated by TSA, which provided conclusive evidence against a clinically relevant impact of CTLA-4 rs231775 on the risk of acute renal graft rejection under the allelic (G vs A), dominant (GG/AG vs AA) or the recessive model (GG vs. AG/AA). Furthermore, none of the positive findings detected in the subsequent subgroup and sensitivity analyses, such as association of the G allele or of the GG genotype with a higher risk in Asian KTRs, were found to be noteworthy by FPRP. Similarly, the application of the FPRP test to statistically significant results of previously published meta-analyses^21–25^ also revealed a lack of noteworthy results in the relationship between CTLA-4 rs231775 and acute renal transplant rejection. Overall, the current findings confute results of the most recent and larger meta-analysis^25^, which however included only 2,581 kidney transplant recipients.

As a key regulator of the immune response magnitude, CTLA-4 genetic variation has been placed at the center of attention by investigators also for a possible role in autoimmunity^48^ and cancer^49^. Results from traditional meta-analyses show that the GG genotype of rs231775, which is associated to lower CTLA-4 expression and hence to a higher T cell activation and proliferation, may confer susceptibility to development of autoimmune diseases, such as rheumatoid arthritis^50^, Hashimoto’s thyroiditis^51^ and myasthenia gravis^52^. On the other hand, individuals with higher expression of membrane CTLA-4, due to the rs231775 A allele, may be at risk of developing multiple types of cancer^53^. These and other evidence support the hypothesis that only an optimal CTLA-4 expression can ensure a state of self-tolerance^54^, being rs231775 G (the low-activity allele) and rs231775 A (the high-activity allele) of CTLA-4 associated, respectively, with susceptibility to autoimmunity and cancer. However, the relevance of CTLA-4 rs231775 has not been consistently reported in every disease condition and is even less pronounced in cancer than in autoimmune diseases^54^. Interestingly, a meta-analysis with TSA have provided convincing evidence for association of CTLA-4 rs231775 with Hashimoto's thyroiditis^55^, nevertheless further investigation is still needed to clarify the role of CTLA-4 rs231775 in different autoimmune disorders and cancer types. In this regard, future application of TSA and/or FPRP to updated meta-analyses could be of value for conclusive demonstration of CTLA-4 rs231775 as susceptibility risk factor for autoimmunity and cancer.

Despite strengths of the present meta-analysis, such as the use of TSA and FPRP, our findings should be interpreted in the light of the following limitations and considerations. First, we attempted to conduct a comprehensive systematic review to identify all potential relevant articles, nevertheless corresponding authors of some eligible publications were unable, or unavailable, to provide genotype distribution of CTLA-4 rs231775. Among these, corresponding authors of two genome-wide association studies (GWASs)^56,57^, reporting no evidence of association between CTLA-4 rs231775 and acute renal graft rejection, were unavailable to provide genotypes for the rs231775 SNP. Therefore, our pooled estimates must be interpreted with caution given the lack of inclusion of all available studies in the meta-analysis. Nevertheless, our TSA results showed that sufficient cumulative evidence has been reached to conclusively exclude a clinically relevant association of the G allele or GG genotype of CTLA-4 rs231775 with a higher risk of acute renal graft rejection. Second, it should be noted that a funnel plot asymmetry, indicating potential publication bias, was indeed detected for the allelic model (G vs. A), which however included only 13 cohorts compared to the 15 cohorts available for the recessive model (GG vs. AG/AA). In spite of this, the statistical correction for this bias, by using the trim and fill method, confirmed the combined risk estimate towards a null effect. Third, the majority of KTRs comprised in the present meta-analysis was of Caucasian ancestry (3,937 out of 5,401), therefore a clinically relevant impact of CTLA-4 rs231775 in other ethnic groups cannot be formally excluded. Fourth, given the large heterogeneity among studies in terms of immunosuppressive therapy, we were unable to evaluate the impact of rs231775 on the risk of acute renal rejection according to a specific immunosuppressive drug. Fifth, we cannot exclude that CTLA-4 polymorphic variants other than rs231775 might play a role as genetic predictive factors for acute renal rejection. In this regard, it should be noted that a number of meta-analyses have been published on the association between other polymorphisms of CTLA-4 and acute renal rejection^20–22,58,59^, however none of these studies applied TSA or FPRP for convincing evidence of an association between these additional CTLA-4 SNPs and acute renal allograft rejection. Finally, the lack of informative data in the identified studies precluded the possibility to adjust ORs for clinical confounding factors, and to investigate interaction effects of rs231775 with other polymorphic gene variants of CTLA-4.

In summary, findings of this systematic review and meta-analysis exclude a role of CTLA-4 rs231775 as a genetic risk factor for acute renal transplant rejection. This conclusion is strengthened by results of TSA, which provided conclusive evidence against a clinically relevant impact of CTLA-4 rs231775 on the risk of acute renal graft rejection in overall populations. Nevertheless, large studies comprising Asian or African kidney transplant recipients are still required to clarify the impact of CTLA-4 rs231775 on acute rejection risk in non-Caucasian populations. In addition, investigation is also warranted in kidney transplant recipients to evaluate whether CTLA-4 rs231775 may have an effect on the risk of acute rejection when analyzed in combination with other CTLA-4 gene variants.

## Supplementary information

Supplementary file1 (PDF 112 kb)
