# Supplementary material for: CTLA-4 rs231775 and risk of acute renal graft rejection: an updated meta-analysis with trial sequential analysis
Source: Sci Rep. 2020 Jul 30;10:12850. doi: 10.1038/s41598-020-69849-4 (PMC7393166; doi:10.1038/s41598-020-69849-4)
Supplement: Supplementary file 1 — Supplementary file1 (PDF 112 kb) [file 41598_2020_69849_MOESM1_ESM.pdf]

CTLA-4 rs231775 and risk of acute renal graft rejection: an updated meta-analysis with trial sequential analysis

Sarah Cargnin[1], Ubaldina Galli[2], Jae Il Shin[3], Salvatore Terrazzino[1*]

[1]Department of Pharmaceutical Sciences and Interdepartmental Research Center of Pharmacogenetics and Pharmacogenomics (CRIFF), University of Piemonte Orientale, Novara, Italy; sarah.cargnin@uniupo.it (S.C.); salvatore.terrazzino@uniupo.it (S.T.)

[2]Department of Pharmaceutical Sciences, University of Piemonte Orientale, Novara, Italy; ubaldina.galli@uniupo.it (U.G.)

[3]Department of Pediatrics, Yonsei University College of Medicine, Seoul, Republic of Korea; shinji@yuhs.ac (J.I.S.)

SUPPLEMENTARY MATERIAL

Table S1. Other characteristics of studies included in the meta-analysis of association between CTLA-4 rs231775 and acute renal allograft rejection.

First Author[ref]	Year	Location	Case number (AR/NAR)	Immunosuppressive drugs	Diagnostic criteria of acute rejection	Genotyping method
Dmitrienko[42]	2005	Canada	100 (50/50)	CsA/Tac + AZA/MMF + PDN	Clinical, laboratory and biopsy findings	PCR-RFLP
Gendzekhadze[17]	2006	Venezuela	63 (30/33)	CsA + MMF + PDN	Laboratory and biopsy findings	PCR-RFLP
Gorgi[43]	2006	Tunisia	70 (31/39)	-	-	PCR-RFLP
Wiśniewski[44]	2006	Poland	91 (38/53)	-	Clinical, laboratory and biopsy findings	PCR-RFLP
Haimila[13]	2009	Finland	678 (109/535)	CsA/Tac + AZA/MMF + MPDN	Clinical and biopsy findings	MALDI-TOF
Kim[45]	2010	Korea	325 (59/266)	Tac-based regimen in 37% of treated pts	Clinical, laboratory and biopsy findings	PCR and sequencing
Kusztal[46]	2010	Poland	314 (102/212)	CsA/Tac + AZA/MMF + PDN	Laboratory and biopsy findings	SSP-PCR
Domański[14]	2012	Poland	269 (70/199)	CsA/Tac + AZA/MMF + steroids	Clinical, laboratory and biopsy findings	RT-PCR
Gao[15]	2012	China	167 (45/122)	CsA/Tac + MMF + PDN	Clinical, laboratory and biopsy findings	PCR and sequencing
Canossi[18]	2013	Italy	72 (37/35)	CsA/Tac + MMF	Clinical, laboratory and biopsy findings	SBT
Misra[16]	2014	India	190 (36/154)	CsA/Tac + everolimus/MMF + PDN	Clinical, laboratory and biopsy findings	PCR-RFLP
Ruhi[19]	2015	Turkey	81 (34/47)	CIs/m-TOR Is + MMF + PDN	Biopsy proven	PCR-RFLP
Niknam[29]	2017	Iran	172 (45/127)	CsA + MMF + PDN	Clinical, laboratory and biopsy findings	PCR-RFLP
Oetting[30]	2019	USA	2872 (492/2380)	steroids, CIs and/or Tac	Clinical, laboratory and biopsy findings	SNPs Array

Abbreviations: AR, acute rejection group; AZA, azathioprine; CIs: calcineurin inhibitors, CsA, cyclosporine; MMF, mycophenolate mofetil; MPDN, methylprednisone; m-TOR Is, m-TOR inhibitors; NAR, no acute rejection group; PCR, polymerase chain reaction; PCR-RFLP, polymerase chain reaction-based restriction fragment length polymorphism; PDN, prednisone; pts, patients; rATG, rabbit anti-thymocyte globulin; RT-PCR, real-time polymerase chain reaction; SBT, sequence-based typing; SSP-PCR, sequence specific primers polymerase chain reaction; Tac, tacrolimus.

Table S2. Assessment of Study Quality.

First author/ year	Quality indicators from Newcastle-Ottawa Scale								
	**Selection (0-4)**				**Comparability (0-2)**	**Outcome (0-3)**			**Score (0-9)**
	**1**	**2**	**3**	**4**	**1**	**1**	**2**	**3**	**Total**
Dmitrienko S (2005)	**1**	**1**	**1**	**1**	**2**	**1**	**0**	**1**	**8**
Gendzekhadze K (2006)	**1**	**1**	**1**	**1**	**0**	**1**	**0**	**1**	**6**
Gorgi Y (2006)	**0**	**0**	**1**	**1**	**0**	**0**	**0**	**1**	**3**
Wiśniewski A (2006)	**1**	**1**	**1**	**1**	**0**	**1**	**0**	**1**	**6**
Haimila K (2009)	**1**	**1**	**1**	**1**	**2**	**1**	**0**	**1**	**8**
Kim HJ (2010)	**1**	**1**	**1**	**1**	**2**	**1**	**0**	**1**	**8**
Kusztal M (2010)	**1**	**1**	**1**	**1**	**0**	**1**	**1**	**1**	**7**
Domański L (2012)	**1**	**1**	**1**	**1**	**0**	**1**	**1**	**1**	**7**
Gao J (2012)	**1**	**1**	**1**	**1**	**2**	**1**	**0**	**1**	**8**
Canossi A (2013)	**0**	**0**	**1**	**1**	**0**	**1**	**0**	**1**	**4**
Misra MQ (2014)	**1**	**1**	**1**	**1**	**1**	**1**	**0**	**1**	**7**
Rui C (2015)	**1**	**1**	**1**	**1**	**2**	**1**	**0**	**1**	**8**
Niknam A (2017)	**1**	**1**	**1**	**1**	**1**	**1**	**0**	**1**	**7**
Oetting WS (2019)	**1**	**1**	**1**	**1**	**2**	**1**	**1**	**1**	**9**

NEWCASTLE - OTTAWA QUALITY ASSESSMENT SCALE COHORT STUDIES
Wells, G. A, Shea, B., O'Connel, D. et al. The Newcastle-Ottawa scale (NOS) for assessing the quality of nonrandomised studies in meta-analyses. http://www ohri ca/programs/clinical_epidemiology/oxford htm 2009 Feb 1

Note: A study can be awarded a maximum of one star for each numbered item within the Selection and Outcome categories. A maximum of two stars can be given for Comparability

Selection
<u>1) Representativeness of the exposed cohort</u>
a) truly representative of kidney transplant recipients in the community*
b) somewhat representative of kidney transplant recipients in the community*
c) selected group of users eg nurses, volunteers
d) no description of the derivation of the cohort

<u>2) Selection of the non-exposed cohort</u>
a) drawn from the same community as the exposed cohort*
b) drawn from a different source
c) no description of the derivation of the non-exposed cohort

<u>3) Ascertainment of exposure</u>
a) secure medical record*
b) structured interview*
c) written self-report
d) no description

<u>4) Demonstration that outcome of interest was not present at start of study</u>
a) yes*
b) no

Comparability
<u>1) Comparability of cohorts on the basis of the design or analysis</u>
a) study controls for HLA matching*
b) study controls for any additional factor*

Outcome
<u>1) Assessment of outcome</u>
a) histology/laboratory findings*
b) record linkage*
c) self-report
d) no description

<u>2) Was median or mean follow-up long enough for outcomes to occur</u>
a) yes (at least 1 year)*
b) no

<u>3) Adequacy of follow up of cohorts</u>
a) complete follow up - all subjects accounted for*
b) subjects lost to follow up unlikely to introduce bias - small number lost < 20% to follow up, or description provided of those lost) *
c) follow up rate > 20% (select an adequate %) and no description of those lost
d) no statement
